# Sick Leave within 5 Years of Whiplash Trauma Predicts Recovery: A Prospective Cohort and Register-Based Study

**DOI:** 10.1371/journal.pone.0130298

**Published:** 2015-06-22

**Authors:** Tina Birgitte Wisbech Carstensen, Per Fink, Eva Oernboel, Helge Kasch, Troels Staehelin Jensen, Lisbeth Frostholm

**Affiliations:** 1 The Research Clinic for Functional Disorders and Psychosomatics, Aarhus University Hospital, Aarhus, Denmark; 2 Department of Neurology, Aarhus University Hospital, Aarhus, Denmark; 3 Danish Pain Research Center, Aarhus University Hospital, Aarhus, Denmark; University of Geneva, SWITZERLAND

## Abstract

**Background:**

10–22% of individuals sustaining whiplash trauma develop persistent symptoms resulting in reduced working ability and decreased quality of life, but it is poorly understood why some people do not recover. Various collision and post-collision risk factors have been studied, but little is known about pre-collision risk factors. In particular, the impact of sickness and socioeconomic factors before the collision on recovery is sparsely explored. The aim of this study was to examine if welfare payments received within five years pre-collision predict neck pain and negative change in provisional situation one year post-collision.

**Methods and Findings:**

719 individuals with acute whiplash trauma consecutively recruited from emergency departments or primary care after car accidents in Denmark completed questionnaires on socio-demographic and health factors immediately after the collision. After 12 months, a visual analogue scale on neck pain intensity was completed. 3595 matched controls in the general population were sampled, and national public register data on social benefits and any other welfare payments were obtained for participants with acute whiplash trauma and controls from five years pre-collision to 15 months after. Participants with acute whiplash trauma who had received sickness benefit for more than 12 weeks pre-collision had increased odds for negative change in future provisional situation (Odds Ratio (OR) (95% Confidence Interval (CI) = 3.8 (2.1;7.1)) and future neck pain (OR (95%CI) = 3.3 (1.8;6.3)), controlling for other known risk factors. Participants with acute whiplash trauma had weaker attachment to labour market (more weeks of sick leave (χ^2^(2) = 36.7, *p* < 0.001) and unemployment (χ^2^(2) = 12.5, *p* = 0.002)) pre-collision compared with controls. Experiencing a whiplash trauma raised the odds for future negative change in provisional situation (OR (95%CI) = 3.1 (2.3;4.4)) compared with controls.

**Conclusions:**

Sick leave before the collision strongly predicted prolonged recovery following whiplash trauma. Participants with acute whiplash trauma had weaker attachment to labour market pre-collision compared with the general population. Neck pain at inclusion predicted future neck pain. Acute whiplash trauma may trigger pre-existing vulnerabilities increasing risk of developing whiplash-associated disorders.

## Introduction

Whiplash trauma is an acceleration-deceleration mechanism of energy transfer to the neck and head from rear-end, side-impact or frontal motor vehicle collision and may be associated with a variety of symptoms, e.g. neck pain, dizziness, and cognitive symptoms. In the acute phase the condition is named acute whiplash-associated disorders (WAD), when symptoms or disabilities remain after six months the condition is defined as chronic and named chronic WAD [1]. The reason why some develop persistent symptoms, despite no objective findings when clinically or radiologically examined, is disputed. The cause seems to be multifactorial, with a variety of biopsychosocial features likely contributing to the development of persistent pain [2,3].

Previous studies in individuals with chronic WAD have found strong associations between poor recovery and pre-collision factors such as back pain [4], unspecified pain condition [5,6], poor general health [7,8], high frequency of attendance to family physician [4], and extensive use of health care [7,9]. Together, these findings raise the possibility that pre-existing vulnerabilities in individual patients may play a role for recovery or lack of such.

Persistent pain and disability following whiplash trauma should be considered as not only an impairment in health-related quality of life, but also as a potential social decline with affected work capability reported in 15–22% of individuals experiencing acute whiplash trauma [5,10]. Leth-Petersen et al. found that persons with chronic WAD had 16–21% lower employment propensity one year post-collision than matched controls in the general population [7]. Within this line of thinking, it is obvious to look into possible social vulnerabilities before the collision. Only few studies have examined the impact of pre-collision socioeconomic factors and the evidence is conflicting. Some studies suggest that low education [3,5,11,12], unemployment [5], and low family income [11] increase the risk of poor recovery, while other studies find no association between poor recovery and prior earnings [7] or low education [13]. Only one study has explored the impact of receiving welfare benefits before the whiplash trauma, e.g. sick pay and disability pension, and found that short term health-related benefits increased the risk of chronic neck pain by 65% while long term health-related benefits and unemployment benefits did not predict recovery [9]. However, this study was based on self-reported questionnaires regarding measurement of exposure to whiplash trauma and welfare benefits and may be biased.

Most studies on whiplash trauma are based on questionnaires which may introduce recall bias, particularly with respect to pre-collision information collected retrospectively. Furthermore, agreement between self-reported welfare benefit and actual received benefit is low for temporary welfare payment such as sickness benefit and unemployment benefit [14]. Scandinavian countries have a tradition of maintaining administrative databases with high-coverage data offering the possibility to investigate pre-collision factors with robust data.

According to a disease model of whiplash, in which pre-collision physical, psychological, and social vulnerabilities are involved in the development of persisting symptoms post-collision, we wished to examine the potential association between pre-collision welfare payment and recovery for individuals with acute whiplash trauma. Therefore we aimed to explore: 1) if people with acute WAD seeking medical care have weaker attachment to labour market before the collision (more weeks of sick listing, unemployment, and social assistance) compared with a matched sample in the general population; 2) if receiving sickness benefit, social assistance, or being unemployed in the five years preceding the collision are associated with negative change in health-related provisional situation and considerable neck pain one year after the collision; 3) if experiencing a whiplash trauma is associated with negative change in health-related provisional situation one year after the collision.

## Material and Methods

### Study Design and Population

This study is a secondary analysis of a large multicenter intervention study of individuals with acute whiplash trauma seeking medical care. They were followed shortly after the collision and one year hereafter [5,15–17]. The present study consists of two separate populations: 1) the cohort of individuals with acute whiplash trauma referred from emergency departments and family physicians and 2) a cohort of controls matched on gender, age and region of residence.

We consecutively recruited individuals who had experienced an acute whiplash trauma from April 2001 to June 2003 from emergency departments or family physicians after motor vehicle collision injuries were sustained in four former counties in Denmark (now two out of five regions in the country). The included were aged 18–70 years, experienced rear-end, side-impact or frontal car collision, experienced WAD symptoms within 72 hours, could understand written and verbal Danish, could be examined within ten days of the collision, and had no alcohol or drug abuse, no fractures or dislocations of the cervical spine (WAD grade 4), no amnesia or unconsciousness in relation to the collision, no injuries other than the whiplash injury, no significant pre-collision physical or psychiatric disorder, and no self-reported average neck pain during the preceding six months exceeding five on a visual analogue scale (VAS) 0–10, where 0 = no pain and 10 = worst possible pain. Participants were included in the study within a median of five days post-collision (q_1_ = 3, q_3_ = 6). Participants signed a written informed consent if they agreed to participate and completed a questionnaire within 10 days after the collision on socio-demographics, neck pain at inclusion, and pre-collision pain. Twelve months after the collision they rated their neck pain intensity for the preceding week. For more details, see other articles from this multicentre study [5,15].

A randomly sampled general population cohort of controls matched on gender, age (± 30 days), and region of residence at the time of collision was identified in the Danish Civil Registration System. Controls could not be 1) in the WAD cohort in this study, or 2) be a control more than once in this study. Controls were sampled in a proportion of five to one case to obtain high statistical power. Data on welfare payment were obtained for each participant with acute WAD and related controls for a period of five years prior to the collision and 15 months after. The rationale for including a general population control cohort in this study was to be able to show the distribution of welfare payment in the general population as we wanted a frame of reference as to the distribution of welfare payment before the collision in the WAD cohort. The general population cohort therefore serves as background data.

The study was approved by the local ethical committees, The Regional Committee on Health Research Ethics of South Denmark, and conducted in accordance with the Helsinki II declaration. Data on WAD and control cohorts retrieved from the Danish Civil Registration System were not anonymized prior to access. Access to these data was approved by The National Board of Health in Denmark, The Regional Committee on Health Research Ethics of South Denmark and The Danish Data Protection Agency.

### Welfare Payment Register

The Danish Register for Evaluation of Marginalization (DREAM) is a database administered by the Danish Ministry of Employment, the Ministry of Social Affairs and Integration and the Ministry of Children and Education [14]. Access to the register requires permission from the Danish Data Protection Agency. At birth, all Danish children receive a Civil Registration System number. DREAM includes all individuals with a Civil Registration System number who have received social benefits or any other welfare payments since July 1991. Persons not registered in the database have not been entitled to any social welfare payments since the database was initiated. Welfare payments comprise sickness benefit, disability pension, unemployment benefit, maternity leave pay, state education fund grants, age retirements etc. A welfare payment is registered in DREAM for a week if the individual has received welfare payment for at least one day during a week. Only sick leave periods exceeding two weeks are included in DREAM as the first two weeks are paid by the employer. Sick leave of more than two weeks is paid by the public sector. If sick leave lasts for more than two consecutive weeks, the first two weeks are also counted.

### Pre-Collision Variables

#### Welfare benefit variables

We included three potential predictive variables comprising 1) sickness benefits, 2) unemployment benefits, and 3) social assistance, all counted as number of weeks of welfare benefits in the five years preceding the collision. The distributions of weeks of all welfare payments were extremely skewed. Subsequently, we grouped the observations based on normative criteria.

#### Sickness benefits

Sickness benefits include all individuals who receive sickness benefits such as employed citizens being ill full or part time, unemployed citizens currently ill, citizens in activation programs currently ill, and citizens in flexible jobs but currently ill. Sickness benefit was categorized according to its duration: 1) No sickness benefit, 2) 1–12 weeks, and 3) more than 12 weeks. In Denmark you could receive sickness benefit for up to two years at that time, and if you were still sick and unable to work after that period, you got transferred to permanent health-related benefit or social assistance. The period of 12 weeks was based on World Health Organisation (WHO)’s definition of chronic illness as the course of an illness lasting more than three months.

#### Unemployment benefits

The unemployment variable consists of all unemployed citizens who receive unemployment benefit (full or part time, during vacation or activation) together with citizens on social assistance who are deemed ready-to-work, only receiving social assistance because of unemployment, but having no entitlement to unemployment benefit. Unemployment benefit was categorized according to its duration: 1) No unemployment, 2) 1–52 weeks, and 3) more than 52 weeks. In Denmark in 2001–2003 it was not possible to receive unemployment benefit for more than four years, and individuals remaining unemployed after that period were transferred to social assistance, which entails lower payment. The cut off at 52 weeks was based on Organisation for Economic Co-operation and Development (OECD)’s definition of long-term unemployment as that involving people out of work and looking for work for 12 months or more.

#### Social assistance

Social assistance is a welfare payment administered by the municipal social service department and is allocated if citizens are unable to support themselves. Excluded from this variable are citizens on social assistance deemed ready-to-work, who are only receiving social assistance because of their unemployment, but who are otherwise not entitled to unemployment benefit. This group falls within the unemployment variable. Social assistance was categorized according to its duration: 1) No social assistance, versus 2) one week or more. The frequency of individuals who had received social assistance in this sample was low therefore to maintain statistical power regarding this variable only two groups were prepared.

### Self-Reported Variables Regarding the WAD Cohort

#### Socio-demographic characteristics

The socio-demographic variables at time of collision included age, gender, and education (‘higher education >4 years’ versus ‘no or other education’).

#### Pre-collision unspecified pain condition

Participants were asked the following question about pre-collision unspecified pain: “Within the last five years, have you suffered from an illness that has caused a persistent pain condition that has entailed absence from work during a consecutive period of two weeks or more? (yes/no)”

#### Neck pain at inclusion

At inclusion participants rated their average neck pain intensity since the collision on a 11-point VAS scale (0 = no pain, 10 = worst possible pain) [18,19].

### Outcome Measures

Outcome was measured in terms of ‘negative change in health-related provisional situation’ and ‘neck pain’. ‘Negative change in health-related provisional situation’ was recorded for both control and WAD cohort. ‘Neck pain’ was measured only for the WAD cohort as there were no data on the controls regarding this outcome measure.

#### Negative change in health-related provisional situation

**Fig 1:**
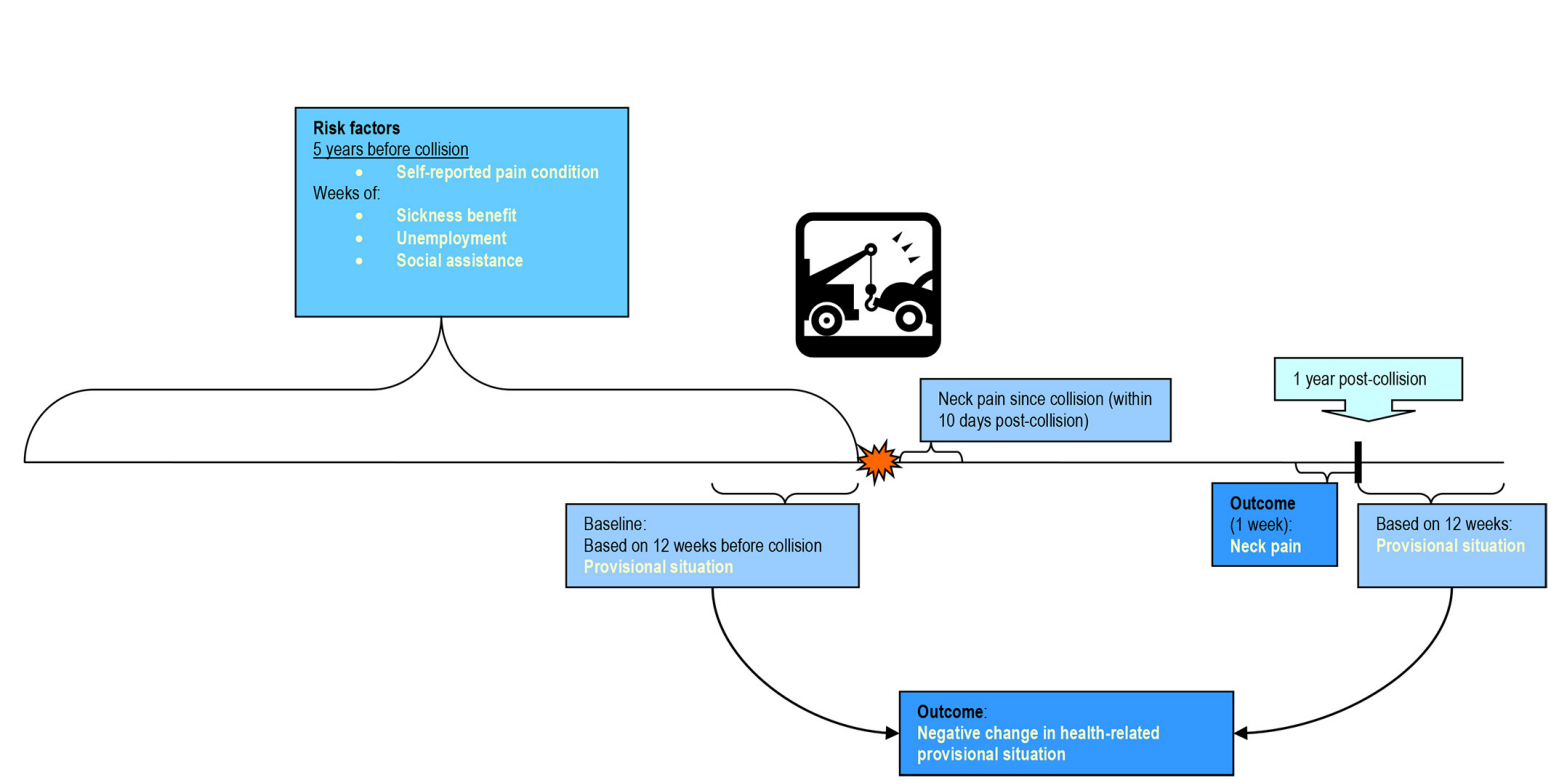
Time line of potential predictive factors and outcome measures.

We categorized provisional situation at two time points: 1) A 12-week period immediately before the collision and 2) a 12-week period from 12–15 months after the collision (Fig 1). Periods of 12 weeks were chosen because the assessment period had to be more than one week as welfare benefits can change several times within a few weeks.

The groups comprised: 1) Age retirement, 2) Self-supporting, 3) Labour-market-related benefits, 4) Temporary health-related benefits, and 5) Permanent health-related benefits (Table 1). These groups were based on the work of Hjollund et al. [14] with the exception that we discriminated between temporary and permanent health-related benefits to render visible possible differences in duration of health disability. Temporary health-related benefits included all individuals who had received sickness benefits (except sickness benefit from flexible job) together with vocational rehabilitation benefits. Permanent health-related benefits included anticipatory pension scheme, light jobs, disability benefits, flexible jobs, sickness benefits from flexible job, unemployed after flexible jobs, voluntary early retirement scheme from flexible jobs. Table 1 describes welfare payments.

**Table 1:** Categorization of provisional situation.

Provisional situation:	Age retirement	Self-supporting	Labour-market-related benefit	Temporary health-related benefit	Permanent health-related benefit
Welfare payment	Voluntary early retirement scheme^a^	No welfare payment	Unemployment benefit	*Sickness benefit* ^*i*^	*Flexible job* ^*j*^
	Public retirement pension^b^	State Education Fund grants	Subsidized employment^d^	*Vocational rehabilitation benefit*	*Unemployed after flexible job*
		Maternity leave pay	Social assistance^e^		*Anticipatory pension scheme*
		Leave-of-absence schemes	*Unemployment benefit during vacation—from unemployment*		*Light job* ^*k*^
		*Adult Education Fund grants* ^*c*^	*Introductory benefit* ^*f*^		*Sickness benefit from flexible job*
		*Unemployment benefit during vacation—from employment*	*Start help* ^*g*^		*Voluntary early retirement scheme from flexible job*
			*Ready-to-work welfare benefit* ^*h*^		*Disability benefit* ^*l*^

Added codes or codes changing groups differing from the original grouping of Hjollund et al. are written in italics. For details on the specific coding refer to the authors.

^a^ Persons who have been members of an unemployment insurance fund for the requisite number of years are entitled to retire at the age of 60 and receive a national benefit comparable to unemployment benefit.

^b^ A national benefit paid to all Danish citizens from the age of 65 (before 1999: 67 years).

^c^ Employed adults over 25 years of age taking leave from job to get an education. Payment is comparable to unemployment benefit.

^d^ Paid by the Public Employment Service to an employer of a long-term unemployed person.

^e^ Welfare payment administered by the municipal social service department. The amount is based on a calculation of economic needs. The payment normally requires the person seeking job through the Public Employment Service.

^f^ Foreigners with residence permit are entitled to introductory benefit.

^g^ For persons who can not support themselves living in Denmark less than 7 years within the last 8 years.

^h^ Persons receiving social assistance, introductory benefit, start help or subsidized employment and the municipal social service department has evaluated that the person is only receiving this benefit because of unemployment but is not entitled to receive unemployment benefit. The person is therefore deemed available for work.

^i^ A municipally administered payment transferred to the person or the employer, if the employer pays the normal wage to the sick-listed employee.

^j^ Jobs created for persons with limited work capability. The person receives the normal wage and the payment is transferred to the employer.

^k^ Jobs on special terms regarding pay and working hours for citizens receiving anticipatory pension.

^l^ A pay supplement for disabled people who are entitled to anticipatory pension scheme but are still working and thereby supports oneself.

Individuals were categorized into the group in which the majority of the 12 weeks was registered. If an individual had emigrated for all 12 weeks of the registration period, the individual was categorized with missing data. If at least one week of welfare payment codes other than emigration was present, we categorized according to these codes. A hierarchy of groups overruling other groups was created, i.e. if an individual within the 12-week period had one week on age retirement, the individual entered this group even if there were more weeks on other types of benefits (Table 2).

**Table 2:** Hierarchies of the categorization of provisional situation at baseline and one year follow-up.

	Baseline	One year follow-up
1.	Age retirement (one week overrules all other codes)	Permanent health-related benefit (one week overrules all other codes)*
2.	Permanent health-related benefit (one week overrules all other codes, except hierarchy 1)	Age retirement (one week overrules all other codes, except hierarchy 1)
3.	Temporary health-related benefit (> = 6 weeks overrules all other codes, except hierarchy 1 and 2)	
4.	Labour-market-related benefit (> = 6 weeks overrules all other codes, except hierarchy 1, 2 and 3)	
5.	Self-supporting (> = 6 weeks overrules all other codes, except hierarchy 1, 2, 3 and 4)	

*At the age of 65–67 (dependent on when you are born) all citizens in Denmark receive public retirement pension regardless of previous welfare payment. With our outcome measure we intend to register negative change in health status. Therefore we wanted to keep the information on permanent health-related benefit (the person is highly probable still sick) even if the person has retired.

We then made a binary variable in which participants were defined according to: 1) Negative change in provisional situation, and 2) positive or no change in provisional situation from baseline to 12-month follow-up (Table 3). We defined a negative change as follows: If an individual was self-supported or received labour-marked-related benefits at baseline and one year later was receiving temporary health-related benefits, it was defined as a negative change in provisional situation, and if an individual was self-supported, or received labour-marked-related benefits, or temporary health-related benefits at baseline and one year later received permanent health-related benefits, this was also defined as a negative change in provisional situation.

**Table 3:** Categorization of negative change in provisional situation from baseline to 1 year follow-up for WAD and control cohorts.

	Provisional situation one year post-collision				
Provisional situation at baseline	Age retirement	Self-supporting	Labour-marked-related benefit	Temporary health-related benefit	Permanent health-related benefit
**Age retirement**	Neutral	Not possible	Not possible	Not possible	Not possible
**Self-supporting**	Neutral	Neutral	Neutral	*Negative*	*Negative*
**Labour-marked-related benefit**	Neutral	Neutral	Neutral	*Negative*	*Negative*
**Temporary health-related benefit**	Neutral	Positive	Neutral or positive	Neutral or positive	*Negative*
**Permanent health-related benefit**	Neutral	Positive	Positive	Positive	Neutral or positive

#### Neck pain

At 12-month follow-up the individuals in the WAD cohort rated their neck pain intensity during the preceding week on a 11-point VAS scale (0 = no pain, 10 = worst possible pain). Participants were asked: “This is your assessment of your average neck pain during the last week, please tick it off, even if you have no pain”. The pain score was dichotomized on the basis of the work of Collins et al. [20] and is in line with already published articles from this multicenter study [5,15,16,21,22]. Scores from 0–3 were considered as “minimal pain”, and scores from 4–10 were considered “considerable pain”.

### Statistical Analysis

#### General statistics

Crude comparisons on categorical variables were examined using the Pearson´s χ^2^ test and with regard to the continuous variable (neck pain at inclusion) the Mann Whitney´s U test was used. Two-sided *P* values < 0.05 were considered statistically significant. The statistical program used for the analysis was STATA 11 [23].

#### Multiple regression models

Multiple logistic regression analysis was performed for each of the two outcome measures at 12-month follow-up as dependent variables to further investigate potential association when including other predictive variables. To avoid over-fitting, 10 to 15 cases for each explanatory parameter should be estimated [24,25]. The selection of potential explanatory variables was made a-priori based on our hypotheses, which build upon rational and theoretical choices and previous research, and we made a priority list. Regarding negative change in provisional situation, the number of cases allowed 5–7 parameters in the model, therefore pre-collision sickness benefit, unemployment, social assistance, gender, and age could be included. As regards neck pain, the number of cases allowed 12–18 parameters in the model and therefore all variables on the priority list could be included (pre-collision sickness benefit, unemployment, social assistance and pre-collision pain condition together with gender, age, education, and neck pain at inclusion). Age and neck pain at inclusion were entered as continuous variables.

Furthermore, a multiple logistic regression was performed for negative change in provisional situation at 12-month follow-up as dependent variable including both cohorts in the analysis to investigate if sustaining a whiplash trauma predicted future negative provisional situation. The number of cases allowed 11–17 parameters in the model, therefore cohort affiliation, pre-collision sickness benefit, unemployment, social assistance, gender, and age could be included in the analysis.

Results are presented as odds ratios (OR) with 95% confidence intervals (CIs). To investigate model fit, we used the Hosmer-Lemeshow fit statistic [26] for discrimination of the area under the Receiver Operating Characteristic curve (ROC) [27] Area under the Receiver Operating Characteristic curve (AUC) [25,28] and a heuristic shrinkage estimate [29]. A check for collinearity in the models has been performed using correlation matrix of coefficients.

## Results

### Participation

**Fig 2:**
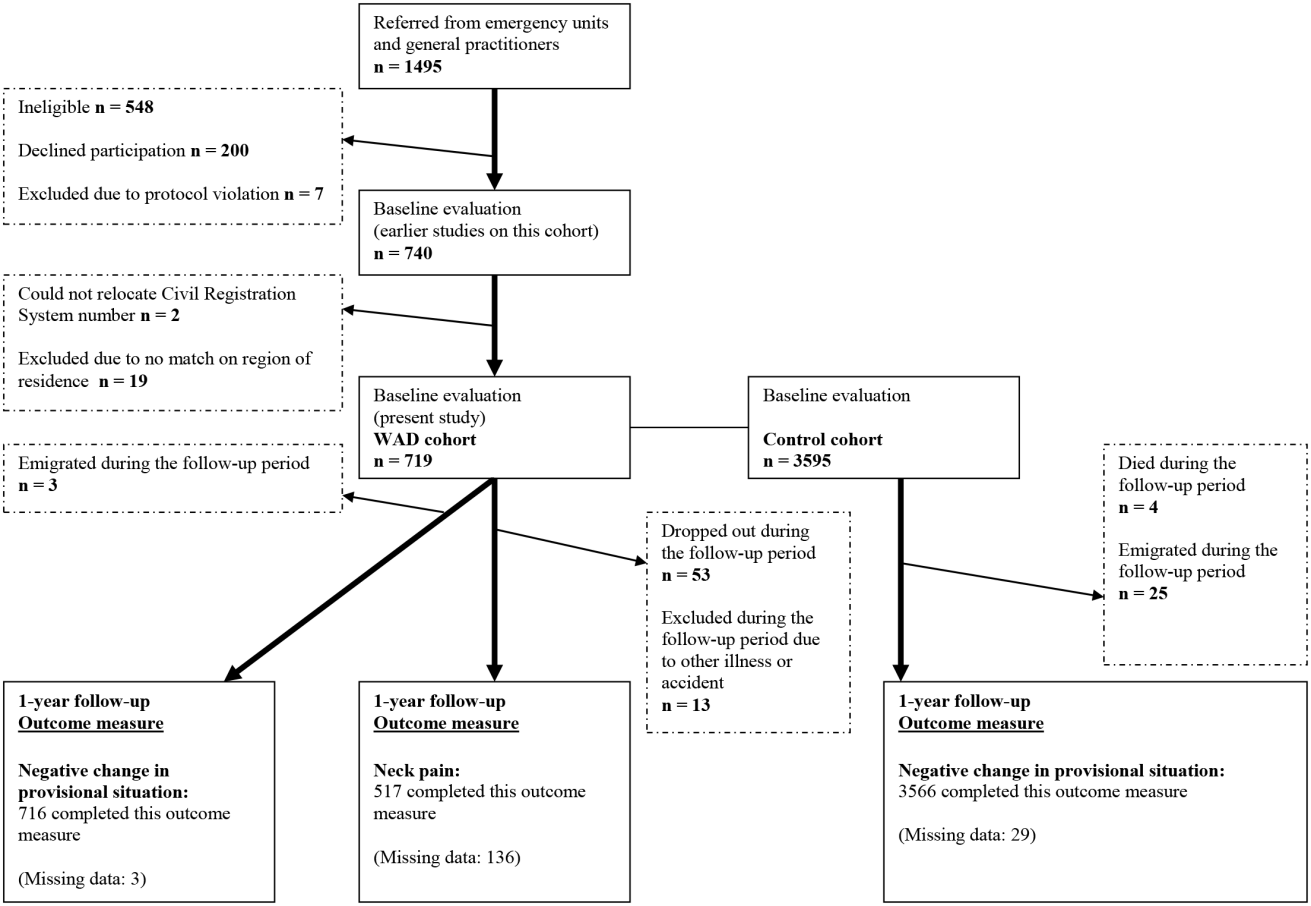
Flow chart of inclusion, exclusion and outcome of whiplash and control cohorts.

1,495 individuals with acute whiplash trauma were recruited (898 women, 597 men). Initially 740 subjects joined the study of which 19 were not residents in a Danish municipality at the time of the collision, and the Civil Registration System number of 2 participants could not be obtained. 755 were excluded for other reasons (Fig 2). The mean age of the included 719 participants with acute WAD was 34.4 years, and 64.4% of the sample was female. Among the 548 subjects ineligible for inclusion the percentage of men was statistically significantly higher than among subjects participating (42% versus 36%) (χ^2^(1) = 4.86, *p* = 0.05). Also, among the 200 subjects declining participation, the percentage of men was statistically significantly higher (49% versus 36%) (χ^2^(1) = 10.8, *p* = 0.001) than among participants. The 53 subjects lost to follow-up did not differ from those completing with respect to gender but were statistically significantly younger (mean age 31.1 versus 35.2) (Mann Whitney’s U Z = 2.5, *p* = 0.01).

3,595 controls were sampled, matched to the participants with acute WAD with regard to gender, age and region of residence.

### 12-Month Follow-Up

716 participants with acute WAD were categorized into the first outcome parameter: Negative change in provisional situation with a coverage of 99.6% as 3 persons emigrated during the follow-up year.

At 12-month follow-up, 517 individuals from the WAD cohort rated their neck pain (response rate: 72%); 185 (36%) reported considerable neck pain. The 136 non-completers were younger than the completing sample (Mann Whitney’s U Z = 3.5, *p* = 0.0005) (mean age 32.0 versus 36.0) and more likely to be male (45% versus 33%) (χ^2^(1) = 6.7, *p* = 0.009).

### Raw Comparison

#### Participants with acute WAD versus General Population Controls

Participants in the WAD cohort did not differ from controls with respect to weeks of social assistance during the five years before the collision or provisional situation at baseline (Table 4). However, the individuals in the WAD cohort had more sickness benefit in the five years preceding the collision than controls in the general population (χ^2^(2) = 36.7, *p* < 0.001), 19.5% of the WAD cohort versus 12.1% of controls received sickness benefit of more than 12 weeks during the five years before collision. Furthermore, individuals with acute WAD had a higher degree of unemployment during the five years preceding the collision than controls (χ^2^(2) = 12.5, *p* = 0.002), 15.0% in the WAD cohort versus 11.5% controls being unemployed for more than a year during the five years preceding the collision.

**Table 4:** Characteristics of WAD and control cohorts, and WAD cohort split into positive or no change versus negative change in provisional situation.

							Change in provisional situation for patients	N = 716	Differences between positive or no change versus negative change		
		WAD cohort	Control cohort	Differenses between patients and controls			Positive or no change	Negative change			
**Pre-collision measures:**		N = 719	N = 3595				N = 646	N = 70			
		n (%)	n (%)	**χ** ^**2**^	**df**	***p***	n (%)	n (%)	**χ** ^**2**^	**df**	***p***
*Welfare benefits 5 years back*:											
Sickness benefit (%)	**1: No sickness benefit**	400 (55.6)	2369 (65.9)	36.7	2	**<0.001**	372 (57.6)	25 (35.7)	27.3933	2	**<0.001**
	**2: 1–12 weeks**	179 (24.9)	792 (22.0)				164 (25.4)	15 (21.4)			
	**3: >12 weeks**	140 (19.5)	434 (12.1)				110 (17.0)	30 (42.9)			
Unemployment (%)	**1: No unemployment benefit**	405 (56.3)	2260 (62.9)	12.5	2	**0.002**	368 (57.0)	34 (48.6)	6.8573	2	**0.032**
	**2: 1–52 weeks**	206 (28.7)	923 (25.7)				188 (29.1)	18 (25.7)			
	**3: >52 weeks**	108 (15.0)	412 (11.5)				90 (13.9)	18 (25.7)			
Social assistance (%)	**1: No social assistance**	609 (84.7)	3111 (86.5)	1.7	1	0.192	552 (85.5)	54 (77.1)	3.3510	1	0.067
	**2: >1 week**	110 (15.3)	484 (13.5)				94 (14.6)	16 (22.9)			
*5 years pre-collision self-reported*:											
Unspecified pain condition (%)	**Yes**	169 (23.5)	—	—	—	—	138 (21.4)	31 (44.3)	17.7434	1	**<0.001**
	**Unaccounted**	9 (1.3)	—				9 (1.4)	0			
**Baseline measures:**											
Provisional situation at baseline (%)	**Age retirement**	7 (1.0)	37 (1.0)	8.5	4	0.076	7 (1.1)	0	3.44	4	0.487
	**Self-supporting**	587 (81.8)	2832 (78.8)				525 (81.3)	59 (84.3)			
	**Labour-marked-related benefit**	78 (10.9)	429 (11.9)				69 (10.7)	9 (12.9)			
	**Temporary health-related benefit**	27 (3.8)	115 (3.2)				25 (3.9)	2 (2.9)			
	**Permanent health-related benefit**	20 (2.8)	182 (5.1)				20 (3.1)	0			
	**Unaccounted**	0.0	0.0				0	0			
No or other education (%)	**Higher education > 4yrs**	60 (8.3)	—	—	—	—	57 (8.8)	3 (4.3)	1.9843	1	0.159
	**No or other education**	614 (85.4)	—				546 (84.5)	66 (94.3)			
	**Unaccounted**	45 (6.3)	—				43 (6.7)	1 (1.4)			
								z	*p*
Mean neck pain at inclusion, VAS (SD)	**Accounted**	4.4 (2.0)	—	—	—	4.2 (2.0)	5.7 (2.3)	-5.210	**<0.001**
	**Unaccounted N = 3, mean (SD)**	3.3 (2.3)	—	—	—	—	—		

df = degrees of freedom

Statistically significantly more individuals with acute WAD (n = 70, 10%) compared with controls (n = 100, 3%) had negative change in provisional situation one year after the collision (χ^2^(2) = 77.5, *p* < 0.001).

#### Negative change in provisional situation in the WAD cohort

Individuals with acute WAD experiencing negative change in provisional situation one year after the collision had received more weeks of sickness benefit during the five years preceding the collision; 42.9% had received sickness benefit for more than 12 weeks pre-collision versus 17.0% for positive or no change in provisional situation (χ^2^(2) = 27.4, *p* < 0.001). They had also received more unemployment benefit; 25.7% had been unemployed for more than a year during the five years preceding the collision versus 13.9% for positive or no change in provisional situation one year after the collision (χ^2^(2) = 6.9, *p* = 0.032). Furthermore, more individuals with acute WAD with negative change reported the presence of prior pain condition within five years of the collision, 44.3% versus 21.4% for positive or no change in provisional situation (χ^2^(1) = 17.7, *p* < 0.001) and more individuals with acute WAD with negative change experienced greater neck pain at inclusion (5.7 versus 4.2; Mann Whitney’s U Z = -5.210, *p* < 0.001). Individuals with acute WAD with negative change in provisional situation one year after the collision did not differ from individuals with acute WAD with positive or no change in provisional situation with respect to education, provisional situation at baseline, or social assistance during the five years preceding the collision (Table 4).

### Multiple Logistic Regression Models

#### Negative change in provisional situation

We set up a multiple logistic regression model regarding the WAD cohort including gender, age, pre-collision sickness benefit, unemployment, and social assistance. Table 5 shows that the odds for negative change in provisional situation one year after the collision were nearly four times as high for individuals who had received sickness benefit for more than 12 weeks during the five years preceding the collision than for individuals having no sickness absence during that period (OR (95% CI) = 3.81 (2.05; 7.06), *p* < 0.001) adjusted for remaining explanatory variables. Receiving unemployment benefit and social assistance within the last five years before the collision did not predict negative change in provisional situation and there was no statistically significant association between gender and negative change in provisional situation. Evaluating model fit the Hosmer-Lemeshow fit statistic showed: χ^2^(8) = 10.50, *p* = 0.23, the AUC: 0.66, and the Heuristic shrinkage estimate: 0.75. A check for collinearity in the model has been performed using correlation matrix of coefficients. The test did not indicate problems with collinearity.

**Table 5:** Multiple logistic models on the WAD cohort with odds ratios of potential predictive variables on provisional situation and considerable neck pain at 12-month follow-up.

	Negative change in *provisional situation* ^*a*^ *n = 716*		Considerable neck pain^b^ *n = 476*	
	OR (95%CI)	P value	OR (95%CI)	P value
1. Age (older age)	1.01 (0.99; 1.03)	0.446	1.01 (0.99; 1.03)	0.275
2. Female gender	0.90 (0.52; 1.56)	0.715	**2.11 (1.28; 3.49)**	**0.003**
3. Sickness benefit				
1: No sickness benefit	1.00	-	1.00	-
2: 1–12 weeks	1.32 (0.67; 2.63)	0.425	1.14 (0.66; 1.97)	0.648
3: >12 weeks	**3.81 (2.05; 7.06)**	**<0.001**	**3.34 (1.77; 6.32)**	**<0.001**
4. Unemployment benefit				
1: No unemployment benefit	1.00	-	1.00	-
2: 1–62 weeks	0.72 (0.38; 1.39)	0.334	0.93 (0.54; 1.62)	0.806
3: >62 weeks	1.22 (0.62; 2.43)	0.563	0.85 (0.43; 1.65)	0.589
5. Social assistance				
1: No social assistance	1.00	-	1.00	-
2: >1 week	1.91 (0.97; 3.77)	0.061	0.52 (0.23; 1.14)	0.102
6. Pre-collision pain condition	**—**	**—**	**2.43 (1.43; 4.12)**	**0.001**
7. Neck pain at inclusion	—	—	**1.48 (1.31; 1.67)**	**<0.001**
8. < 4 years of higher education	—	—	1.00	-
> 4 years higher education	**—**	**—**	0.44 (0.18; 1.07)	0.072

^a^ Negative change in provisional situation (n = 70, 10%) compared to positive or no change in provisional situation (n = 646, 90%).

^b^ Considerable neck pain (n = 167, 35%) compared to minimal neck pain (n = 309, 65%).

#### Neck pain

A multiple logistic regression model for neck pain included pre-collision sickness benefit, unemployment, social assistance, pre-collision pain condition, gender, age, education, and neck pain at inclusion was performed for the WAD cohort. As shown in Table 5, receiving sickness benefit for more than 12 weeks during the five years preceding the collision increased the odds for considerable neck pain one year after the collision by more than three times compared with individuals having no sick leave (OR (95% CI) = 3.34 (1.77; 6.32), *p* < 0.001) adjusted for remaining explanatory variables. The odds for considerable neck pain were more than twice as high for women than for men (OR (95% CI) = 2.11 (1.28; 3.49), *p* = 0.003), pre-collision unspecified pain increased the odds for considerable neck pain 12 months after the collision (OR (95% CI) = 2.43 (1.43; 4.12), *p* = 0.001), and neck pain at inclusion increased the odds for considerable neck pain 12 months after the collision (OR (95% CI) = 1.48 (1.31; 1.67), *p* < 0.001) adjusted for remaining explanatory variables. There was no statistically significant association between neck pain at 12-month follow-up and age, education, prior social assistance, or prior unemployment. Evaluating model fit the Hosmer-Lemeshow fit statistic showed: χ^2^(8) = 11.70, *p* = 0.17, the AUC: 0.80, and the Heuristic shrinkage estimate: 0.92. A check for collinearity in the model has been performed using correlation matrix of coefficients. The test did not indicate problems with collinearity.

#### Exploring exposure to whiplash trauma

We set up a multiple logistic regression model for negative provisional situation at 12-month follow-up including both cohorts. The model comprised cohort affiliation (WAD or control cohort), gender, age, pre-collision sickness benefit, unemployment, and social assistance. We found that individuals experiencing whiplash trauma had more than tripled the odds for negative change in provisional situation compared with controls (OR (95% CI) = 3.13 (2.25; 4.35), p < 0.001) adjusted for remaining predictive variables. Several variables statistically significantly increased the odds for negative change in provisional situation in the sample of the two cohorts altogether; older age (OR (95% CI) = 1.02 (1.01; 1.04), p = 0.003), sickness benefit 1–12 weeks pre-collision (OR (95% CI) = 2.03 (1.33; 3.09), p < 0.001), sickness benefit >12 weeks pre-collision (OR (95% CI) = 5.42 (3.66; 8.03), p < 0.001), social assistance (OR (95% CI) = 2.11 (1.39; 3.22), p < 0.001), unemployment benefit >52 weeks (OR (95% CI) = 1.60 (1.06; 2.42), p = 0.026). There was neither statistically significant association between gender and negative change in provisional situation nor between unemployment benefit 1–52 weeks and negative change in provisional situation. Variables other than cohort affiliation may only be interpreted as control for confounding in this model, given the combination of two very different samples. Evaluating model fit the Hosmer-Lemeshow fit statistic showed: χ^2^(8) = 9.48, *p* = 0.30, the AUC: 0.78, and the Heuristic shrinkage estimate: 0.96.

## Discussion

In this prospective study we found that number of weeks on sick leave in a period of 5 years before an acute whiplash trauma were strongly associated with poor outcome: Receiving accumulated sickness benefit of 12 weeks or more before the collision were associated to both negative change in provisional situation and considerable neck pain one year after the collision controlled for other known risk factors. Moreover, we found that the WAD cohort had more weeks of sick leave and unemployment before the collision compared with a matched general population sample. Finally, as expected, the impact of experiencing a whiplash trauma increased the likelihood for future negative change in provisional situation compared with controls in the general population.

This study is, to our knowledge, the first to investigate pre-collision socioeconomic factors in a cohort of individuals with acute WAD recruited from the health care sector. The study included individuals with acute WAD recruited from emergency units and family physicians as opposed to studies including claimants, where litigation issues may introduce bias. Obvious selection bias due to socio-demographic characteristics was minimized as health care in Denmark is free of charge for all citizens. Furthermore, data was obtained from a Danish national public register on social benefits and any other welfare payments and therefore considered robust without missing data in terms of participants declining participation or non-completed questionnaires and no information-, recall-, or response bias. Another methodological strength is the matched-control group applied to evaluate if the WAD cohort differs from the general population (matched on gender, age, and region of residence). Methodological strengths of the questionnaire-based part of the study are the sample size and high response rates. This allowed us to employ multiple statistics with concurrent inclusion of several variables and a higher control for confounding.

The study has several limitations.

1. The study group may not be representative of all subjects that have sustained an acute whiplash trauma as we recruited our study subjects from individuals who sought medical care (consulting an emergency unit or a family physician). As women have a higher frequency of visits to physicians, according to statistics Denmark, (a mean of 13 visits for women versus 9 visits for men in 2012), women may also be more likely to consult a physician when experiencing symptoms after acute whiplash trauma. Furthermore, more men than women were excluded and declined participation in this study and more than one third of all recruited were initially excluded from the study; one fourth of those due to inclusion later than ten days after the collision, and nearly one fifth had injuries other than the whiplash trauma.
2. Less than two weeks of sick leave is not registered in the database as these weeks are paid by the employer. However, if sick leave lasts for more than two consecutive weeks, the first two weeks are also counted. Short time sick leave may therefore be underestimated in this study. Moreover, we do not know the reason for sick leave, it may vary considerably from cancer, pain conditions, and psychological distress to a broken leg and we have not looked into possible patterns of sick leave which might hold interesting information.
3. Participants with average neck pain of >5 on a box scale during the last six months pre-collision were excluded due to this study being part of an intervention study. 51 participants have been excluded on this basis. Such exclusion may have introduced systematic bias as you would expect previous neck pain to make you more likely to experience negative change in provisional situation. However, the bias is in the direction of underestimating the impact.
4. Neck pain is not an optimal endpoint because it is a common symptom in the general population ranging from 20–40% [30]. It is a weakness of the study that it was not possible to retrieve information about neck pain in the controls. However, to date there is no accepted definition of recovery [2,31], and in this study we included a work- and health-related outcome (change in health-related provisional situation) as well as a health-related outcome (neck pain).
5. The lack of psychological measures known to predict poor prognosis, such as post traumatic stress symptoms and catastrophizing, is a limitation in this study. E.g. baseline measures of catastrophizing were not available.
6. The self-reported measures represent a limitation in this study as self-reported measures may introduce recall bias. These count education, pre-collision unspecified pain condition, neck pain at inclusion, neck pain at 12-month follow-up.
7. The statistical accuracy of the models varies. Regarding the model examining risk factors for considerable neck pain at 12-month follow-up, the model fit seems adequate (AUC: 0.80). Regarding the model examining negative change in provisional situation including both cohorts, the accuracy of the model also seems adequate (AUC: 0.78). However, the model fit for negative change in provisional situation in individuals with acute WAD is weaker as the AUC is 0.66. Nevertheless, the focus of this article is merely to investigate potential risk factors and not to establish a predictive model.

More than 3 months of sick leave prior to the collision predicted negative change in provisional situation and considerable neck pain one year after the collision. Being unemployed or receiving social assistance before the collision did not predict poor recovery in this study. It seems to be that social exclusion is not the issue here but merely health related vulnerability before the collision. Another recent study similarly found that receiving short term health-related benefits (sick pay and rehabilitation benefits) nearly doubled the odds (OR = 1.92) of subsequent chronic WAD and that long term health-related benefits and unemployment benefits did not increase risk [9]. In that study socioeconomic benefits and exposure to whiplash trauma were self-reported and therefore holds possibility for recall-, information-, or response bias. Furthermore, welfare payment status were a snapshot in time not accumulated over the years. However, our results resemble their findings of short term health-related benefits being a strong predictive factor also when robust register data are used and the predictive value can be shown on self-reported (neck pain) and register-based outcome measures (negative change in provisional situation) alike.

We found that having unspecified pain before the collision adds risk of experiencing neck pain after the collision with an odds ratio of 2.43. This is also found in another study with a Relative Risk of 2.1 [6]. Thus, a prior pain condition increasing the probability for developing a pain condition again if exposed to acute pain. This can be due to sensitisation as previously reported in individuals with WAD [32–34] and also found in post-operative patients [35–37]. A genetic contribution is reported in a few prospective studies on whiplash injuries, and similar painful conditions [38,39], where persons with high pain sensitive haplotypes of COMT-alleles carry a raised risk for long-term disability after whiplash injuries, but also risk for development of other so-called idiopathic pain disorders as TMD/TMJ (Temporomandibular disorders/Temporomandibular joint disorders) and fibromyalgia. Furthermore, we found that individuals with acute WAD seeking health care were more likely to be socially marginalized before the collision than controls in the general population. That is 7.4 percentage points more individuals with acute WAD than individuals in the general population reported more than three months of prior sick leave (RD (95% CI) = 7.4% (4.3%; 10.5%), p < 0.0001) and 3.6 percentage point more individuals with acute WAD than in the general population were unemployed for more than a year preceding the collision (RD (95% CI) = 3.6% (0.7%; 6.4%), p = 0.0074). One might speculate that prior poor general health may lead to an increased risk of sustaining a whiplash trauma or reporting the presence of symptoms and seeking treatment when experiencing a whiplash trauma. A plausible explanation of high sick leave before the collision for individuals with acute WAD may be that persons earlier exposed to illness entailing sick leave may have different help seeking behaviours when experiencing new symptoms i.e. seek medical care and report sick to a higher degree than the general population, a behaviour they may have been prone to do before the collision when experiencing symptoms. Prior pain condition and sick leave before the collision may be two sides of the same coin, representing vulnerability before the collision covering biological (e.g. biomarkers, genetics), psychological (e.g. perception of and reaction to symptoms) and social factors (e.g. social support and attachment to labour market).

Social marginalization for the WAD cohort is also shown after the collision, as 10% of the individuals with acute WAD had negative change in provisional situation one year after the collision versus 3% of the general population which corresponds with another Danish study reporting that persons with chronic WAD had 16–21% lower employment propensity one year post-collision than matched controls in the general population [7]. We found that initial neck pain increased the risk of future neck pain (OR = 1.48). This is in line with previous research finding initial pain levels consistently predicting poor prognosis [3,40]. In this study we found odds for negative change in provisional situation between 2.3 to 4.4 for individuals with acute WAD compared with the general population. Moreover, several pre-collision factors predicted poor recovery in this study. Persons who have experienced health problems or long-term unemployment during the years before the collision may have fewer resources to cope with the acute pain and strain following a whiplash trauma, increasing the risk of acute symptoms developing into chronic pain. We propose the hypothesis that the collision is a triggering factor for predisposing factors to ignite the development of health disabilities.

Our study supports the hypothesis that individuals experiencing negative illness trajectory and reduced work capability after whiplash trauma have weaker attachment to the labour market before the collision compared with the general population and recovered individuals suggesting multifactorial vulnerability before the collision to be predisposing for persistent pain triggered by the actual whiplash trauma. Thus, two individuals experiencing similar motor vehicle collisions may react differently according to prior strengths or vulnerability. We will note that while this study points to the possible role of psychosocial determinants before the collision as risk factors for development and maintenance of persistent pain or lack of recovery other factors are also likely to play a role. For example genetic and biological mechanisms related to the processing of nociception may also represent risk factors for the development of pain and lack of recovery.

Our results add to the understanding of social as well as psychological and physical pathways of developing persistent pain following acute whiplash trauma and highlight the importance of a greater focus on pre-collision characteristics such as provisional situation.
